# Research progress in the regulation of endothelial cells and smooth muscle cells using a micro–nanostructure

**DOI:** 10.1186/s12938-025-01337-0

**Published:** 2025-01-23

**Authors:** Songhao Liu, Juan Yan, Mengyu Gao, Hongxia Yang

**Affiliations:** 1 Northwest Institute of Plateau Biology, Chinese Academy of Sciences, Xining, 810008 China; 2 School of Energy and Electrical Engineering, Qinghai University, Xining, 810016 Qinghai China; 3 Department of Pharmacy, Medical College of Qinghai University, Xining, 810016 China

**Keywords:** Micro–nanostructure, EC, SMC, Stent

## Abstract

Recently, the incidence rate and mortality of various acute or chronic vascular occlusive diseases have increased yearly. As one of the most effective measures to treat them, vascular stents have been widely studied by researchers, and presently, the most commonly used is a drug-eluting stent, which reduces the process of rapid endothelialization because the drug is not selective. Fortunately, with the discovery and exploration of micro–nanostructures that can regulate cells selectively, reducing the incidence of "intravascular restenosis" and achieving rapid endothelialization simultaneously are possible through a special structure that cannot only improve endothelial cells (ECs), but also inhibit smooth muscle cells (SMCs). Therefore, this paper mainly introduces the preparation methods of micro–nanostructures used in the past, as well as the detection methods of EC and SMC. Then, the various functions of different dimensional structures for different cells are summarized and analyzed. Finally, the application of micro–nanostructure in future stent materials is summarized and proposed.

## Introduction

In recent years, various vascular obstruction problems have been severe. This is not just true for the elderly but also for those with bad habits who put heavy pressure on their blood flow, which can lead to angina pectoris, stroke, myocardial infarction, etc. [1, 2]. The direct reason for this is that the excessive accumulation of plaque around the arterial wall disturbs the blood flow, while the basic reason is generally that some diseases change the physical conditions, including shear stress, pulsation, and tension in the blood vessels, which leads to endothelial cell (EC) and smooth muscle cell (SMC) dysfunction [3–6]. At present, the main treatment schemes are drug therapy, surgical bypass therapy, and percutaneous coronary intervention (PCI). Although drug therapy is relatively safe and stable, it often fails to achieve ideal results for the elderly and patients with severe diseases. Therefore, it is necessary to consider the patient's adaptability issues [7]. Surgical bypass therapy can only treat blood circulation problems for a while, but it cannot prevent the further development of coronary atherosclerosis and has not been widely used because of the difficulty of surgical operation. PCI includes balloon dilation and stent implantation, but balloon dilation can only alleviate the disease at the initial stage of vascular stenosis and has a high incidence of restenosis. The worst problem is that the elasticity of the blood vessel itself gradually makes the temporary expansion ineffective. Due to the excellent functionality of only inflicting small wounds and the exceptional therapeutic effect of vascular stents, stents have been widely studied and are the first choice for patients [7–9]. The first application was a bare metal stent (BMS), which solved preliminary vascular blockage successfully, but the mechanical damage caused by surgery and long-term retention in the body caused a series of inflammatory reactions [10]. Due to these issues, the SMC that controls vascular contraction undergoes abnormal proliferation and migration and differentiates into the synthetic phenotype, which causes the extracellular matrix (ECM) to excessively secrete, forming the dissimilation and proliferation of the vascular wall and leading to restenosis [6, 11, 12].

In order to solve this adverse reaction, several drugs, such as rapamycin and paclitaxel, have been introduced as drug-eluting stents (DESs) to inhibit the proliferation of SMC, which reduces the probability of restenosis. However, these drugs have no selection, inhibiting the recovery of EC, delaying re-endothelialization, and hindering treatment [13, 14]. Although there are such issues, the incidence of restenosis has been reduced to a relatively low level. Therefore, the following target focuses on biodegradable stents, which can promote the recovery of EC via excellent biocompatibility to solve the chronic inflammation caused by long-time retention. Further, the rapid recovery of EC itself will inhibit the proliferation of SMC to reduce the occurrence of restenosis [15]. However, the accumulation of degraded substances caused by the time and uniformity of degradation may also lead to blockages in other places [7, 16]. In addition, its long-term efficacy is still controversial due to limited clinical research, causing its application to be infrequent, and DES remains the primary choice for patients.

Therefore, to address the issues of DES drug selectivity and permanent retention, studies have demonstrated that the growth of SMCs and ECs is the key to solving the problem [17, 18]. In 1911, Harrison found that embryonic frog cells grew along spider silk, proving that the surface topography of the material affects the cell's behavior [19]. Further, various composite micro–nano structure materials were used for cell research, which determined the impact of morphology on cell proliferation, adhesion, differentiation, etc. [20, 21]. Some special structures initially promoted or inhibited the growth of EC and SMC [10]. Therefore, applying a micro–nanostructure to a vascular stent to achieve rapid endothelialization and inhibit the proliferation of SMC is considered a feasible scheme that can bypass the drawbacks of drugs [22].

To date, methods such as soft lithography, photolithography, and electrospinning, have been used to fabricate micro–nanostructures, and chemical deposition, surface plasma treatment, and other processes have been used for superficial modification to adjust the biocompatibility, wettability stiffness, etc., in cell experiments. These experiments have attempted to determine the relationship between the cell response and micro–nanostructures so as to facilitate the design and application of micro–nanostructures [9, 23]. However, the basic theory of the interaction between the cell and structure is not clear when examining various cell types and materials, structures, sizes, and complex reactions of cells [21, 24, 25]. The only preliminary conclusion is that actin and integrin have roles in transmitting and transducing material structural signals, especially in the cytoskeleton [26–28].

Although this theory is not detailed enough for us to directly design the micro–nanostructure required for a vascular stent, the methods of structures' preparation, surface modification, and detection [24, 29–31]; the differences in the effects of structural characteristics on cells [24, 32–34]; and the relationship between materials' surface nature and biocompatibility [31] have been summarized and analyzed in many experiments [24, 29–34]. These achievements not only provide effective references for applying micro–nanostructures to vascular stents, but also ensure scientific and practical application in the later stage. Among various possible structures, the groove has been proven to greatly impact the arrangement of cells. At the same time, Kaunas showed that the directional arrangement of EC is closely related to atherosclerosis [35]. Ordered EC are conducive to maintaining and rebuilding normal vasculature, which further shows the promise of using structures to make up for the defects of DES [35–37]. For example, Chen et al. designed a new three-layer vascular graft with directional microgrooves by combining electrospinning technology to promote the directional growth and migration of human umbilical vein endothelial cells (HUVECs) and endothelialization process [38].

Therefore, this study focuses on ECs and SMCs and covers the preparation of micro–nanostructures and cell-detection methods used in EC and SMC experiments so as to provide a reference for future research. Then, the effects of different structures on EC and SMC are classified and summarized. In addition to illustrating the shapes and functions of the micro–nanostructures currently used, the respective findings of each researcher are also briefly explained. Finally, the application of micro–nanostructures in future stent materials is summarized and proposed.

## Preparation method of micro–nanostructures

With the development of science and technology, the precision of preparing micro–nanostructures has been greatly improved, reaching several nanometers. In various cell experiments, the methods, including ion implantation, electroplating, and spraying, are often combined to modify the properties of structures to meet the basic needs of cell experiments because the materials do not have enough biocompatibility. Currently, the preparation methods of micro–nanostructures for EC and SMC are mainly as follows.

### Lithography

**Fig. 1:**
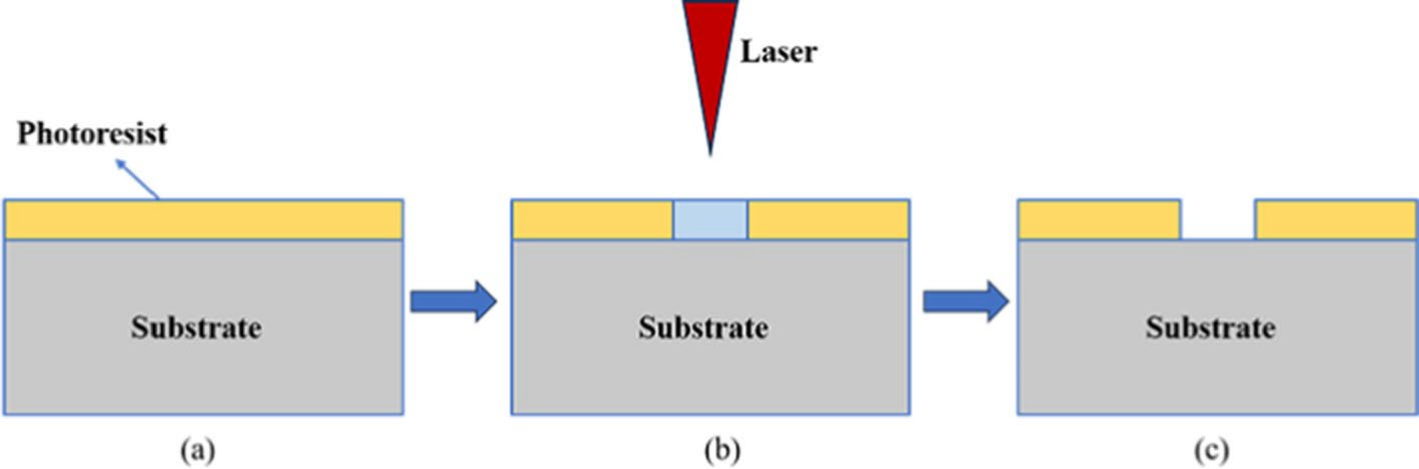
Key processes of electron beam lithography. **a** Spin-coated photoresist, **b** exposure, **c** development

Lithography is a process of exposing a mask coated with specific photoresists, which are optical or electronic-sensitive materials, to make it cross-link and form a computer-prepared pattern on the substrate [39]. According to the type of laser, the technique is mainly divided into ultraviolet, extreme ultraviolet, electron beam, and direct laser writing lithography, and the most commonly used is ultraviolet lithography [40]. The basic step is to remove surface impurities and pollutants to enhance the adhesion with photoresistance through substrate pretreatment, then carry out gluing (Fig. 1a) and pre-drying, whose uniformity and thickness directly affect the quality of the final pattern. The next step is to expose it (Fig. 1b). A mask to block the passage of light to form a specific pattern is not needed if direct laser writing technology is used [41]. Eventually, the target structure can be obtained by developing and fixing it (Fig. 1c) [42].

Although this technique has the advantages of high precision in controlling the shape and size of the pattern and is convenient for removing the photoresist after pattern transfer, the materials are expensive. They must be used on a flat surface for a long time, and extreme cleaning conditions are required in the fabrication process [40]. The quality of the target pattern is affected by the energy and time of exposure, the operation of washing, etc., which need to be optimized and adjusted according to the actual situation.

### Soft lithography

**Fig. 2:**
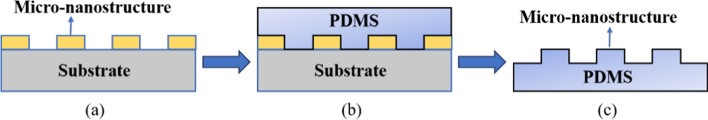
Basic process of soft lithography (PDMS). **a** Preparation template, **b** coating PDMS, **c** demolding

Soft lithography is mainly used to replicate micro–nanostructures, including micro-contact printing, nano-imprint lithography (NIL), and molding. This technology uses elastic materials, especially polydimethylsiloxane (PDMS), to replace the materials prepared using other techniques [43]. The general step is sol preparation, which is selected based on the requirements for material properties, and bubbles are removed to ensure the purity and integrity of the structure. After evenly casting and baking, demolding (Fig. 2) can finally be performed, which damages the integrity of the structure and the regularity of the formwork if the operation is improper [44].

Compared with traditional lithography technology, the accuracy of soft lithography is not limited by the material generally. It can be operated on irregular surfaces and produce complex multi-layer or three-dimensional structures, so it has great advantages in biological experimental research [45–50]. Furthermore, the equipment required is relatively simple, and the production cost is more economical than other techniques. However, this method is prone to structural deformation, even collapse, and cannot obtain deeper structures [43].

### Electrospinning

**Fig. 3:**
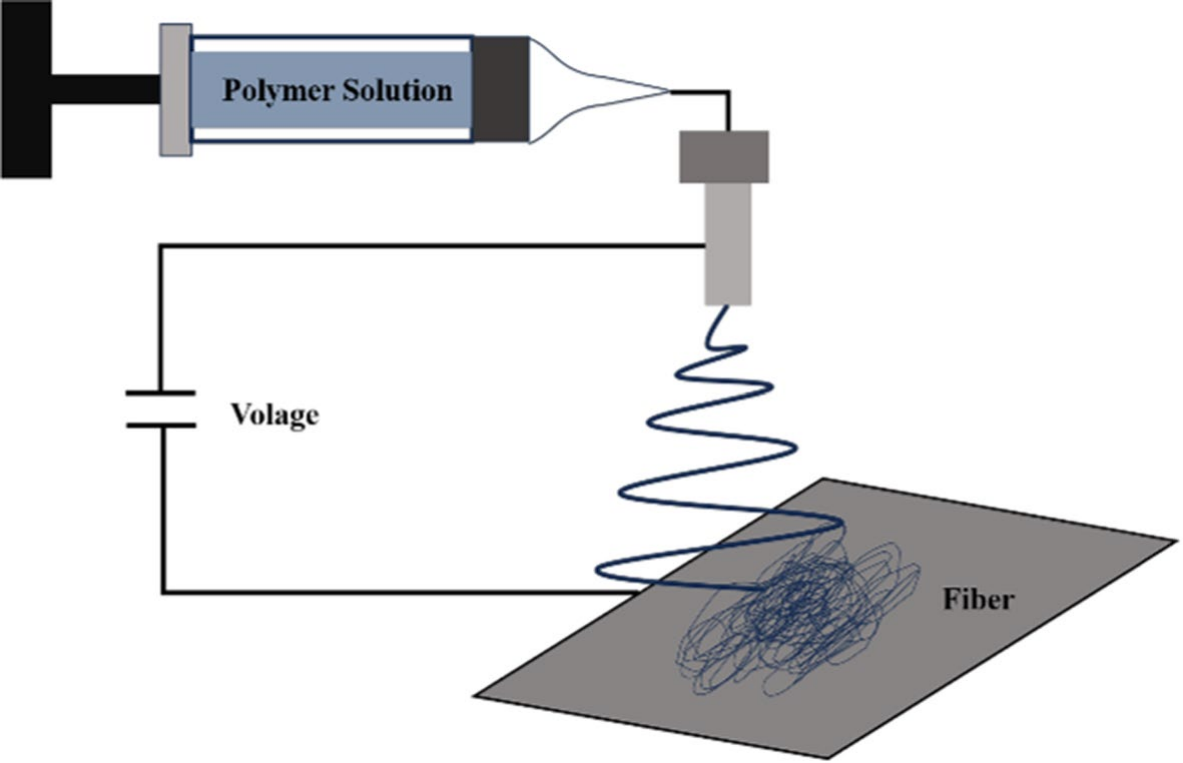
The technological process of electrospinning

Electrospinning cannot directly prepare specific structures but can form nanofibers, a spinning process that uses a polymer solution or melt to form jet flow under a strong electric field [51]. Generally, the nanofibers can be prepared after the solution's preparation by setting the parameters, including the spinning voltage and advance speed (Fig. 3). Although there are no complicated steps, the properties of the prepared solution, such as viscosity and the environmental parameters, have a crucial impact on the uniformity, diameter, and length of the prepared fiber [51, 52].

Optimization can obtain fibers of several nanometers with high porosity and large specific surface areas, which can be combined with different morphological structures. The process is simple and can be produced in large quantities [53]. It has been widely studied in the fields of drug release, wound dressing, filter materials, etc., while the reason it is covered here is that it can be applied on the substrate or used to simulate fibrous tissue and the extracellular matrix [38, 54, 55], which promotes cell proliferation and adhesion. For example, Guo et al. used Poly to simulate the morphology of natural collagen fibrils via electrospinning, which promotes the adhesion and diffusion of EC [56]. Although it is difficult to control the consistency of material size accurately and obtain nanofiber filaments separated from each other, general biological experiments are not limited by these factors [52].

### Others

In addition to the above traditional techniques, chemical etching, hydrolysis electrolytic control, etc., are also used to prepare micro–nanostructures [57–60]. In addition to electrostatic spinning, micro–nano fibers can also be prepared by self-assembly and thermally induced phase separation [61]. Although these methods are simple, convenient, and low-cost, they cannot accurately control the shape and size of the obtained structure directly, so many experiments are required in the early stage to determine the rules. Therefore, in terms of time and accuracy, soft lithography can obtain more perfect results. However, regardless of which method is used, most structural surface properties are changed by chemical coating, deposition, surface plasma modification, and other methods to achieve the experimental purpose.

## Cell test detection

Due to the influence of the micro–nanostructure itself on the instruments or chemicals and the need to study the interaction between the structure and the cell, traditional detection methods must be adjusted. Based on the goals of rapid endothelialization and a low restenosis rate, the proliferation, morphology, arrangement, adhesion, and migration EC and SMC are used as the main testing index. The following section will briefly introduce the detection of these indicators and explain some special experiments.

### Cell proliferation

To promote the proliferation of EC and inhibit the proliferation of SMC, the proliferation intensity of EC and SMC is generally regarded as the first requirement. Common detection methods to determine the proliferation intensity of EC and SMC include 3-(4,5-dimethylthiazol-2-yl)-2,5-diphenyltetrazolium bromide (MTT), Cell Counting Kit-8 (CCK-8), and 5-ethynyl-2’-deoxyuridine (EDU) [38, 59, 62, 63]. For example, Jiang et al. embedded EDU into deoxyribonucleic acid (DNA), dyed the nucleus, and measured the proportion of nucleated cells mixed with EDU using a fluorescence microscope so as to reflect the cell proliferation via DNA activity [63]. In addition, the cytotoxicity kit was also used to stain the living and dead cells and then counted using the corresponding wavelength excitation, respectively, so as to obtain the data on the cell proliferation intensity more comparatively [64]. However, when measuring the absorbance of such methods, it is not only necessary to transfer cells on the structure to a blank porous plate, but also to redesign the parallel control experiment to eliminate the error if the material itself or some coating materials are dissolved in the culture medium.

### Cell adhesion

The cell adhesion strength is usually used to indicate the level of the surficial biocompatibility structure to judge the rationality of the application in a vascular stent. The simplest test method involves washing the unattached cells with phosphate-buffered saline (PBS) and then counting the remaining cells. To ensure the accuracy of the experiment, the blood cell counter is generally used [65, 66]. For example, Peng et al. aspirated the culture medium after different culture times. Then, they harvested the attached cells with a trypsin–EDTA solution and counted them with a blood cell counter so as to show the cell adhesion strength at different stages [59]. In contrast, Shin et al. designed a monocyte adhesion experiment wherein the cells labeled with 3,3′-dioctadecyloxacarbocyanine (DiO) were washed twice with PBS to remove the unbound monocytes. Then, the number of adherent monocytes was calculated by counting the signals labeled with DiO with software [67]. Although there was no significant difference in the accuracy of the two experiments, the latter needed to exclude the influence of DIO introduction through a controlled experiment.

In addition, Potthoff et al. took adhesion measurements of a single HUVEC on different structures by designing a program combining piezoelectric devices, which can measure 16 to 38 cells from each matrix in at least 3 independent samples [68]. Besides these physical methods, Western blot (WB) is often used as a detection method to indirectly reflect the adhesion ability of cells because fibronectin and focal adhesion kinase (FAK) are closely related to cell adhesion. Potthoff obtained the relative intensity of FAK expression through WB, which reciprocally confirmed each other with the adhesive force.

### Cell morphology

The cell morphology reflects the direct impact of the structure on individual cells. On the premise that the base material of the micro–nanostructure is transparent, the optical microscope can be used for rough observation; for example, Sarkar et al. directly used software to measure the size of the long axis and short axis of SMC under an optical microscope that expressed the extent of cell elongation using the aspect ratio [69]. However, most structures still impact light, which greatly reduces the clarity and reliability of the acquired image, so it is necessary to eliminate this effect through filtering or other detection methods [47]. For opaque materials, an optical microscope can do nothing. In order to obtain clear and accurate images, scanning electron microscopy (SEM) is generally used for observation, and cells need to be dehydrated to meet the needs of SEM. Gradient dehydration is generally carried out with alcohol, and ester substances are used to replace alcohol. The critical dryer is used for drying to ensure cell integrity. Finally, the material surface is sprayed with gold to make the material surface conductive so that it can be measured using SEM [64, 70–72].

In addition to the direct observation of cells with precision instruments, the nuclei and skeletons can also be stained for observation through a fluorescence microscope. Not only can the morphology and position of cells be observed, but the basic changes in the nuclei can also be obtained because the nucleus roughly occupies the center of the cell, while the cytoskeleton occupies other space. For example, Thakar et al. stained the cytoskeleton and nucleus, respectively, and performed fluorescence observation and software processing to obtain clear images. They further gained the nuclear volume, projected cell area, and perimeter [60]. The most commonly used nuclear staining agents are hematoxylin, while rhodamine phalloidin is used for the cytoskeleton [73–75]. Although these methods are simple, it is necessary to consider whether the dye interacts with the surface of structures to avoid confusion with the staining cells.

### Cell migration

The speed of EC migration mainly affects the endothelialization process, and some traditional methods generally follow the cells in real-time or delayed tracking through precision instruments after labeling. For example, Biela described the migration results of cells along the x and y directions, respectively, through precise detection [25]. Similarly, calculating the diffusion time corresponding to the change of a certain coverage can also be used to compare the migration degree [47]. In addition, considering the effect of blood flow in vivo, Uttayarat et al. designed a parallel plate flow chamber, which can provide stable laminar shear stress to simulate the actual blood flow affected by the EC labeled with 1,1'-dioctadecyl-3,3,3',3'-tetramethylindocarbocyanine perchlorate (DiI), which can be detected with the delayed fluorescence microscope outside the incubator. In addition, they also used the scratch test to scrape multiple bands with a pipette. They calculated the moving speed by dividing the total distance of cells moving at the edge of the band by the set time point as the control to eliminate the effect of Dil itself [45]. Although the scratch test has the advantages of low cost and easy operation, it has lower accuracy.

Further, Chen designed an interesting experiment similar to the scratch experiment. He covered half of the structure with a sealing film, leaving the remaining half, and inoculated EC. After the EC stably adhered and spread, the sealing film was carefully removed under sterile conditions and gently soaked with PBS. Then, the sample was put back into the original culture medium for further cultivation to be observed under SEM after 48 h and 72 h so as to attain the migration speed [9]. However, the material he used was stainless steel, which has sufficient mechanical strength but damages the integrity of the structure if the material is fragile.

### Cell arrangement

EC and SMC on the inner of blood vessels are longitudinally arranged along the blood vessels in vivo [28, 76]. The directional arrangement of EC and SMC is closely related to atherosclerosis, and their order is conducive to maintaining a normal vascular environment [35]. Therefore, their arrangement is also an important reference for structural function. Because the arrangement of cells needs micro–nanostructure as a specific reference, SEM is usually used for observation or used in immunostaining for fluorescence observation to obtain a more obvious positional relationship. Moreover, most studies have characterized the arrangement of cells by calculating the angle between the long axis of cells and the periodic structure to attain more quantitative results. For example, Uttayarat et al. indirectly stained the actin microfilaments of bovine aortic endothelial cells (BAECs) to observe using confocal microscopy. They showed that the BAECs present a slender shape, and their long axis is "aligned" with respect to the groove within 20°. Meanwhile, the long axis of BAEC and the groove direction between 20° and 90° was defined as "misaligned" [72]. Franco et al. used 45° degrees as the boundary value of cell alignment. At the same time, Sarkar calculated the percentage of cell alignment relative to the structure to indicate the degree of cell alignment [47, 69]. Although they are different indicators defined in the later stage, SEM and fluorescence staining observation are still basically used.

## The regulation of the micro–nanostructure on cells

### Non-periodic micro–nanostructure

**Fig. 4:**
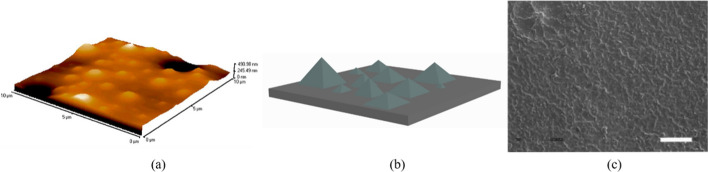
Morphology of non-periodic micro–nanostructures. **a** AFM image of polybromostyrene–polystyrene (PS/PBrS) with nanohills 95 nm high [79]. **b** Pyramid conceptual model. **c** Nanostructured (polylactic-co-glycolic acid) (PLGA) (feature dimensions ≅ 50–100 nm). Original magnification =  × 250; scale bar = 100 μm

At present, the non-periodic micro–nanostructures used include the nano-island, gold tower, and porous structures, and some cannot be described in detail, especially those whose surface reaches micro–nano roughness (Fig. 4). In studying all non-periodic structures, most researchers have mainly focused on cell adhesion, which may be because there are small irregular nanoscale protrusions on the inner wall of blood vessels, which play a role in promoting cell adhesion. The different functions of non-periodic micro–nanostructure are listed in Table 1. Secondly, the environment of ECs and SMCs in the body is filled with nanoscale protein fibers, and the nanoscale roughness is regarded as similar [76, 77]. Although most studies have found that aperiodic structures promote cell adhesion at a certain scale, the pyramid structure prepared by Saux et al. can inhibit EC adhesion, and they believed that this is mainly related to the shape of the pyramid, not to the roughness (Fig. 4b). In addition, they applied arginine–glycine–aspartic acid (RGD) peptide, an integrin ligand, on the material's surface and found that the cell adhesion strength of different concentrations of RGD differed. Combined with the research results of surface chemistry, they concluded that cell adhesion was a two-step process. At the initial stage of contact between the cell and the matrix, topography played a major role. Then, surface chemistry became the decisive factor affecting cell behavior after the initial contact, consistent with the phase intensity of the chemical and physical clues' influence on cells proposed by LIM et al. [57, 78].

**Table 1:** Summary of the non-periodic structures’ properties and the cellular responses they evoked

Island						
Material	Cell type	Height	Diameter	Spacing	Findings	Refs.
PCL PEG	HUVEC	27.4 $nm$	223 $nm$	1638 $nm$	Surface features promote localization in signal transduction pathways. Formation of focal contact and development of cytoskeleton	[80]
PS PBrS	HUVEC	13, 35, 95 $nm$	–	–	The 13 $nm$ high nanohills show significant endothelialization	[79]

Hole				
Material	Cell type	Diameter	Findings	Ref.
Alumina	SMC	20 $nm$ 200 $nm$	200 $nm$ pores contribute to cell elongation and cell cycle, DNA replication, cell proliferation, and signal transduction pathways	[74]

Fiber				
Material	Cell type	Diameter	Findings	Ref.
PCL	HUVEC	200–600 $nm$	A more sensitive signal between FA protein and F-actin transduction may promote cell migration	[56]

Rough surface					
Material	Cell type	Harshness	Height	Findings	Ref.
Si	BAEC	1.1, 1.2, 7.6, 69, 419 $nm$	53, 390, 1858 $nm$	Micron-sized pyramids hinder cell adhesion and migration	[57]

Material	Cell type	Diameter	Findings	Refs.
PGA	SMC	13.0, 12.1, 11.2, 10.0, 8.4, 6.5 $\mu m$	The surface hydrolyzed PGA fibers are a suitable SMC surface	[58]
${SiO}_{2}$	C11STH	6.93, 7.55, 11.06 $nm$	Cells can quickly reshape protein-coating surfaces to meet their needs	[81]
PCL	EC SMC	52–100 $nm$	NaOH treatment enhances cell adhesion and proliferation	[62]
PLGA PU PCL	SMC	21.8, 35.31, 70.30, 206.14 $nm$	The enhancement in cell adhesion is due more to dimensional changes in surface morphological characteristics than surface chemistry or area changes	[65]
PLGA PU	SMC	206.14, 368.82 $nm$	Nanostructures can achieve maximum Bladder SMC function	[66]

*Materials (*PEG* poly (ethylene glycol), *Si* silicon, *PGA* poly (glycolic acid))

*Cells (*HUVEC* human umbilical vein endothelial cell, *BAEC* bovine aortic endothelial cell, *C11STH* human endothelial cell)

As a prerequisite for cells to perform other physiological activities, adhesion plays a particularly crucial role in the healing process following vascular stent implantation. Robust adhesion of ECs facilitates their subsequent proliferation and migration, thereby accelerating the process of re-endothelialization. For example, Buttiglieri et al. designed nano-island structures with different heights by polybromostyrene–polystyrene (PS/PBrS) which showed good endothelialization of 13 nm and 35 nm nanoislands that is related to the secretion of adhesion factors (Fig. 4a) [79]. However, it is not a decisive factor, Nguyen found that there was no significant difference between the 20 nm hole and the 200 nm hole on the adhesion of SMC, but the 200 nm hole had the strongest effect on the elongation of SMC and signal transduction [74]. Therefore, they exhibited the characteristics of stage and mutation and highlighted the importance of size and shape.

In addition, when changing the structural characteristics via coating, ion implantation, etc., the chemical properties inevitably change, affecting the cell's actions. Therefore, Thapa et al. simulated the nano-morphology of natural bladder tissue through cell compatibility materials, changed the characteristic size of the nano-morphology through a chemical treatment to affect the surface area and roughness, and compared it with those without chemical treatment so as to understand the role of independent structures. Using SMC culture, they found that the number of SMCs is significantly increased on submicrostructure poly-ether-urethane (PU) and nanostructured PLGA. Then, PLGA and PU films with normal and nano-surface characteristic sizes were synthesized using a silicone rubber mold to eliminate the influence of chemical factors, which found that the nanostructure PLGA enhanced the number of SMCs. Still, the effect of PU was slightly poor, which indicated the difference in materials [65]. In terms of cell adhesion, they found that the size of PLGA and PU within the nanometer range can improve SMC adhesion and concluded that when adhesion increased with roughness. After eliminating the effect of chemistry via electron spectroscopy for chemical analysis (ESCA), it was assumed that the enhancement in cell adhesion was only due to the size change [65]. Furthermore, the adhesion of PLGA and PCL was higher than PU, which also shows that the nature of the material itself has a certain difference in the impact on the cell. This is why researchers should modify or film the material.

As to why these aperiodic structures affect cell adhesion, Hsu et al. believed that surface features promote the formation of focal contact and the development of a cytoskeleton in the signal transduction pathway. At the same time, Buttiglieri found that the platelet endothelial cell adhesion molecule (PECAM1) and alpha-L integrin can regulate cell behavior, and a micro–nanostructure regulates their secretion [79, 80]. Although the substances studied are different, most of them belong to the category of integrin signal transduction, and this pathway involves far more substances. Therefore, no one can fully explain the change in the whole process. Moreover, more experiments are still needed due to the interwoven chemical and physical signals, as well as the relationship between the roughness and the structural shape.

### One-dimensional periodic micro–nanostructure

**Fig. 5:**
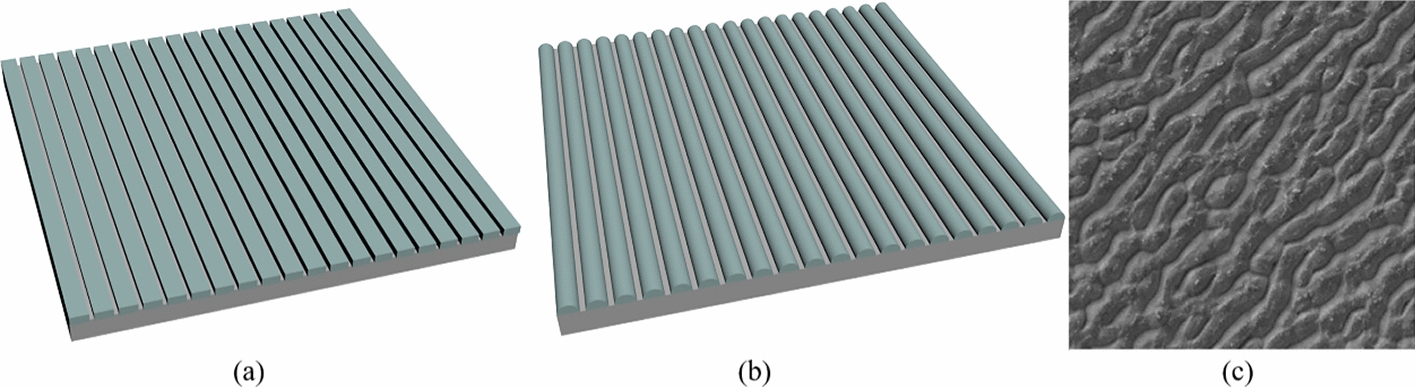
Morphology of one-dimensional periodic micro–nanostructures. **a** Model of the rectangular groove structure. **b** Model of the cylindrical/nanotube groove structure. **c** SEM images of the wall (ripple) structures [83]

At present, the one-dimensional periodic micro–nanostructures used mainly have grooves, nanotubes, and ripple shapes. The groove needs to be subdivided due to the different angles and slopes of the ridge, and they generally promote the proliferation of EC and SMC within a certain size (Fig. 5). Compared with aperiodic and two-dimensional periodic structures, the most obvious effect of a groove is the inevitability of regulating the cell arrangement regardless of the difference in the ridge. For example, Poly glycerol-sebacate (PGS) prepared in a nanoscale groove with an arc-shaped ridge using photolithography and a groove with a rectangular ridge on PDMS prepared using soft lithography can promote the arrangement and elongation of EC. The former reason considered the effect of filo pod substances (Fig. 5a, b) [82]. At the same time, the latter was thought to be due to the terrain affecting the process of cytoskeleton and focal adhesion, affecting the cell arrangement indirectly, but it reduced cell adhesion [10]. Similarly, the nanoscale morphology formed by collagen fibers arranged in parallel can also regulate the arrangement and elongation of EC [70]. It is believed that this is the result of the shape specificity at first. However, two-dimensional periodic microcolumns can also regulate cell arrangement, so detailed reasons must be further explored.

In addition to shape specificity, the regularity of size is interesting. For instance, using soft photolithography, grooves with the same transverse size characteristics and different longitudinal sizes were prepared on a cyclic olefin copolymer (COC) to culture EC. The results showed that a groove structure with a larger longitudinal size promoted the activation of local adhesion kinases in the range of 100 to 1000 nm in immunostaining, while a smaller size promoted the activation of paxillin and caused the aggregation of vinblastin and actin at the adhesion site [68]. These results are a key reminder that the difference in cell response terrain sizes is not necessarily the difference in regulation intensity, but different mechanisms were caused by size differences. Antonini et al. prepared a groove with a constant groove depth (0.4 μm), periodicity, and different ridge sizes using COC. They found that grating with a transverse period of a 2 μm and 1 μm wide ridge was the most suitable for EC expansion. They maximized the mechanical transmission of EC to achieve the best cell arrangement effect by measuring the spread area and body orientation. Considering the anisotropy of the groove, ECs were transfected for analysis, and it was found that the orientation of actin microfilaments indirectly affected the orientation of cells. The influence of these proteins also promoted the maturation of FA, thus promoting the process of FA, while the intensity was affected by the transverse size of the groove [84]. In addition, Sarkar et al. also found that the alignment of SMC did not change significantly with the increase in the groove width in the range of 20–80 μm but affected the aspect ratio of a single cell [69]. This is also a result of the influence of structure on actin arrangement and FA location, which is consistent with the traditional theory that the cell pseudopodium feels the terrain, and integrin transmits signals to the nucleus [85].

In addition, there are fibers in the vascular basement membrane itself, and the existence of fibers makes the inner wall of the blood vessel appear as an irregular submicron groove structure. It is thought that this might play a role in cell arrangement [76, 86]. Therefore, the groove is considered a biomimetic morphology inside blood vessels to some extent. For example, Liang et al. observed that the SMC surface is a nanogroove fiber structure. They prepared a similar structure to imitate the surface morphology of SMC to promote the adhesion, proliferation, and migration of EC to promote the endothelialization process [87]. However, studies directly simulating the fiber structure are still few, and most of them use a fiber structure as a substrate for combination with a structure. For example, using electrospinning and soft lithography to combine the groove micro-pattern with nanofibers can promote the elongation of EC and stimulate its arrangement and diffusion [88]. Hao-Yang Mi used electrospinning to simulate the structure of collagen fibers and the elasticity of elastin, which can achieve the same effect [89]. Although the specific morphology of the nanofiber is not controllable, it still presented groove characteristics overall. In addition, Nozaki et al. directly made a secondary groove with a femtosecond laser. The nanogrooves had a width of 624 ± 9.3 nm and a depth of 102 ± 42 nm, and the microgrooves had a width of 2.0 ± 0.30 μm and a depth of 589 ± 167 nm, which affected the arrangement and adhesion of ECs [90]. Although compared with a simple groove, it does not produce new effects, combining this secondary structure provides more possibilities.

The experiments were conducted in vitro, but the actual ECs and SMCs contacted the complex environment in the blood vessel. Although these experiments cannot fully simulate the three-dimensional internal environment, they still realized the simulation control of some conditions. For example, as mentioned in the detection method, Uttayarat et al. used plasma lithography and ion etching to prepare grooves of different sizes on silicon and assembled a parallel plate flow chamber to simulate the mechanical effect of blood flow, which can guide the rapid initial elongation and arrangement of ECs, while when the parallel plate flow chamber was not working, the microgroove guided the direction of cell migration. The smaller the width of the groove, the more cells migrated laterally, indicating that the physical signals of shear stress and topography interact and compete. That is, when the flow direction is parallel to the groove, it will strengthen the longitudinal migration of cells. When perpendicular to the groove, it will interact with the topographic signals, which are related to the flow speed, reflected through the arrangement of actin and the activity of FAs internally [45]. Considering the interaction between ECs and SMCs, Liu et al. prepared a groove on glass using photolithography, and the nanofibers prepared by electrospinning were attached to the groove to make SMCs penetrate the fiber to grow. At the same time, most of the ECs were concentrated above, similar to the layering relationship in the blood vessel, which can improve the cell viability of the SMCs and orient both ECs and SMCs [48].

It can be seen from the above results that the groove provides a guiding effect for long and straight channels. At the same time, it is not a necessary condition for cell arrangement. Even in the ripple, there is directional cell growth, so the function of the channel is not necessarily related to the shape ridge. The anisotropy of the one-dimensional periodic micro–nanostructure itself is the basic reason for this [83]. The shape of the ridge might only impact the intensity of regulation or the internal mechanism, similarly to the size. In combination with the effect of stress and the interaction of EC and SMC in the above studies, the stiffness, stress, terrain signal, and chemistry interwoven with each other must be considered while applying a vascular stent. This is the key to ensuring the structure can repair the inner wall of blood vessels in vivo and maintain the physiological environment in blood vessels again [5]. Table 2 shows the different functions of the one-periodic micro–nanostructure.

**Table 2:** Summary of the one-dimensional periodic structures’ properties and the cellular responses they evoked

Groove					
Material	Cell type	Height	Period	Findings	Ref.
PGS	BAEC	0.45 $\mu m$	2.5 $\mu m$, 4.1 $\mu m$, 4.5 $\mu m$	Filaments can detect the structural curvature to regulate cell adhesion	[20]

Material	Cell type	Ridge width	Groove width	Height	Findings	Refs.
PDMS	RASMC	–	20, 30, 40, 50, 60 $\mu m$	11 $\mu m$	The arrangement of the cells and actin filaments directly depends on how narrow the passage is	[54]
PDMS	HUVSMC	5 $\mu m$, 12 $\mu m$	20, 50, 80 $\mu m$	5 $\mu m$, 12 $\mu m$	Groove widths from 20 to 80 $\mu m$ have no significant impact on cell arrangement, but they affect cell elongation	[69]
Ti	RAEC	750 $nm$-100 $\mu m$	750 $nm$-100 $\mu m$	–	The cell arrangement increases significantly as the characteristic size decreases	[64]
PDMS	BAEC	5 $\mu m$, 2 $\mu m$	5 $\mu m$ 2 $\mu m$	1.2 $\mu m$	The microchannel substrate induces the spatial positioning of FAs along the groove depth and arranged parallel to the groove with actin microfilaments	[45]
PDMS	HAVSMC	–	10 $\mu m$	–	Cell elongation regulates the SMC proliferation rate	[75]
PDMS	HCAEC HCASMC	–	2, 3, 5 10 $\mu m$	50, 100, 200 $nm$	The lateral spacing decreases and the cell orientation increase as the depth of the groove increases	[25]
Collagen type I	VSMC	330 $nm$	330 $nm$	100 $nm$	Cells maintain the SMC phenotype	[46]
COC	HUVEC	1 $\mu m$ 5 $\mu m$	1 $\mu m$ 5 $\mu m$	0.1–2 $\mu m$ 0.1, 1 $\mu m$	Increased orientation of cells along gratings with deeper grooves	[47]
PDMS	HDMEC	30 $\mu m$	30 $\mu m$	10 $\mu m$	The anisotropy of micropattern channels guides cell elongation. Actin cytoskeleton and focal adhesion	[70]
PELA	HUVEC HUASMC	200 $\mu m$, 300 $\mu m$	100 $\mu m$	–	Terrain cues guide the alignment of SMC and its ECM production	[48]
COC	HUVEC	1000 $nm$	1000 $nm$	100, 400, 1000 $nm$	EC adhesion ranges from 100 to 1000 $nm$	[68]
PDMS	SMC	–	183.3–243.8 $nm$	–	The dominant direction of FA mature adhesion determines the direction of cell adhesion	[49]
COC	HUVEC	0.5–2.0 $\mu m$	0.5–2.0 $\mu m$	400 $nm$	The pattern indicates local adhesion maturation, thereby reshaping the cytoskeleton of the cell	[84]
PLLA	HUVEC	–	9.4 $\mu m$ 7.6 $\mu m$	13.9 $\mu m$ 12.5 $\mu m$	Cells can fill the grooves or change their expanded morphology to accommodate the grooves	[67]
PDMS	HCAECs	–	2 $\mu m$, 10 $\mu m$	200, 350, 650 $nm$	The orientation of the stress fibers is preferably aligned parallel to the groove direction	[71]
PDMS	BAEC	5 $\mu m$ 2 $\mu m$ 0.7 $\mu m$	5 $\mu m$ 2 $\mu m$ 0.7 $\mu m$	1 $\mu m$ 1 $\mu m$ 0.25 $\mu m$	EC morphology and orientation are driven by the positioning and aggregation of FA	[85]
PDMS	BAEC	3.99 $\mu m$ 3.46 $\mu m$ 3.32 $\mu m$ 5.51 $\mu m$	3.85 $\mu m$ 3.73 $\mu m$ 3.62 $\mu m$ 2.65 $\mu m$	0.19 $\mu m$ 0.49 $\mu m$ 0.98 $\mu m$ 4.59 $\mu m$	Changes in depth can effectively induce EC arranging and extending parallel to the channel direction	[72]
PCL	HUVEC	–	116.39 $\mu m$	–	The directional micropattern structure promotes HUVEC migration, alignment, and proliferation	[38]
Nitinol	PEC	2.0 $\mu m$, 624 $nm$		102 $nm$, 589 $nm$	EC prioritizes the recognition of nanopatterned arrays	[90]

Nanotubes				
Material	Cell type	Aperture	Findings	Refs.
TiO2	BAEC MOVAS	30 $nm$	Nanotubular surfaces may preferentially enhance EC growth They reduce SMC proliferation	[59]
TiO2	EC, ECL HCBEPC	15, 20, 30, 50, 70, 100 $nm$	The size of the 15–20 $nm$ spacing supports cell adhesion	[60]

Corrugation					
Material	Cell type	Period	Height	Findings	Ref
PET	HMEC	200 $nm$ 350 $nm$ 300 $nm$	25 $nm$ 50 $nm$ 30 $nm$	The ability of topology to initiate endothelial b-catenin signaling	[83]

*Materials (*Ti* titanium, *PELA* Poly (DL-lactide)–poly (ethylene glycol), *PLLA* poly(L-lactide), *PET* polyethylene terephthalate)

*Cells (*RASMC* rat aortic smooth muscle cell, *HUVSMC* human umbilical vein smooth muscle cell, *RAEC* rat aortic endothelial cell, *HCAEC* human coronary artery endothelial cell, *HCASMC* human coronary artery smooth muscle cell, *HDMEC* human dermal microvascular endothelial cell, *HUVEC* human umbilical vein endothelial cell, *HUASMC* human umbilical artery smooth muscle cell, *PEC* porcine endothelial cell, *MOVAS* mouse aortic vascular smooth muscle cell, *ECL* endothelial cell line, *HCBEPC* human cord blood endothelial progenitor cell, *HMEC* human microvascular endothelial cell)

### Two-dimensional periodic micro-nanostructure

**Fig. 6:**
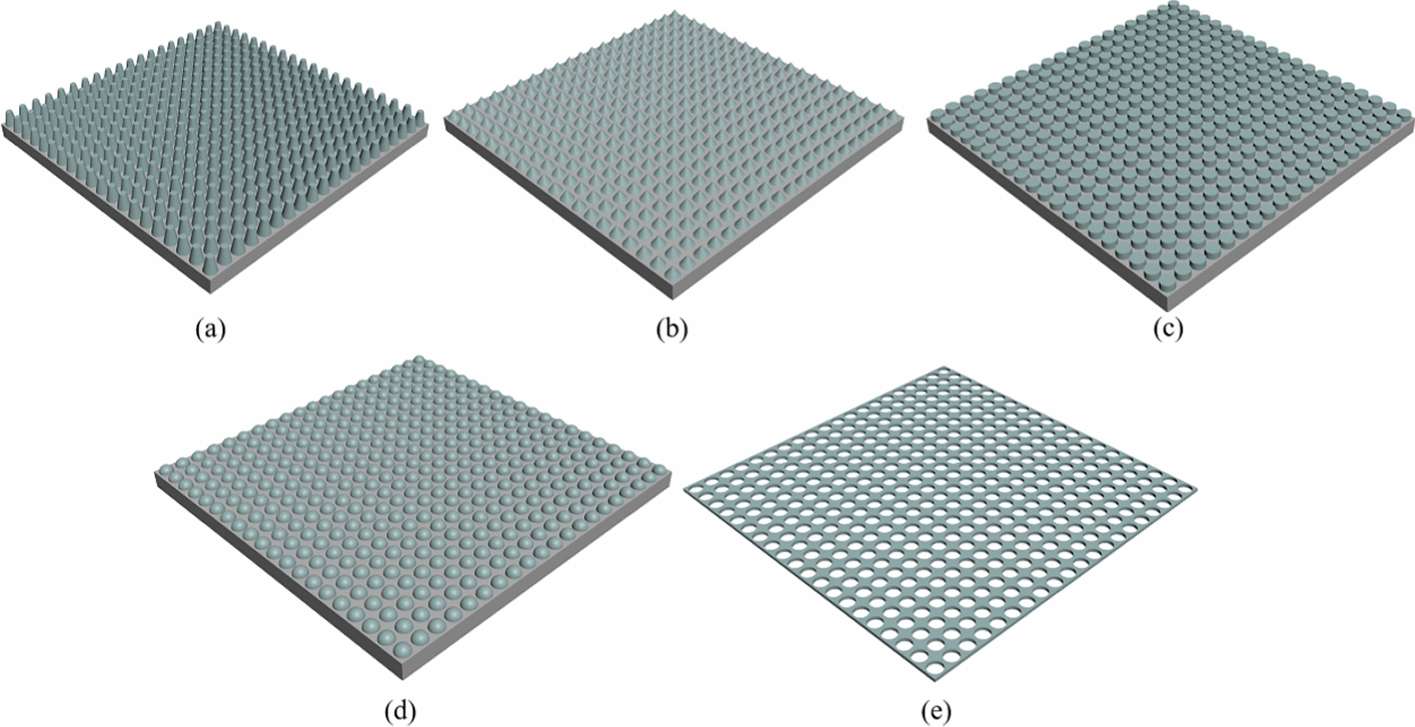
Morphology of two-dimensional periodic micro–nanostructures. **a** Model of round-shaped structure (wide bottom and narrow top). **b** Model of cone structure. **c** Model of cylinder structure. **d** Model of hemisphere structure. **e** Model of hole structure

Two-dimensional periodic micro–nanostructures include nanoislands, columns, cones, hemispheres, and pore-shaped structures (Fig. 6) and are introduced in Table 3. In addition to a pore-shaped structure, which is mainly used to study the relationship between signal transmission and pore diameter in the hierarchical culture of cells (Fig. 6e), other structures focus on the relationship between the trend of the cell with size, and most structures with a smaller vertical height and unit size are more prone to cell adhesion and growth [91]. However, Dickinson et al. found that the same microcolumn structure reduced EC adhesion when the substrate was silica when height was greater than 3 nm, while EC elongation and alignment were enhanced if the substrate was PDMS (Fig. 6c) [92]. Therefore, they argued that the elongation and arrangement of ECs are jointly regulated by the morphology and stiffness of the matrix, while the stiffness is a part of biocompatibility [93, 94].

**Table 3:** Summary of the two-dimensional periodic structures’ properties and the cellular responses they evoked

Island				
Material	Cell type	Height	Findings	Ref.
PS, PBrS	HGTFN	13 $nm$, 35 $nm$, 95 $nm$	13 $nm$ island produces the largest cellular response	[95]

Hole				
Material	Cell type	Diameter	Findings	Ref.
SiO SiO2	EC	2 $\mu m$, 4 $\mu m$	Porous tubes promote the production of endothelial monolayers	[91]

Pillar						
Material	Cell type	Diameter	Spacing	Height	Findings	Ref.
SiO2	EC HUVEC	1–5.6 $\mu m$	0.6–15 $\mu m$	1, 3, 6, 8 $\mu m$	The elongation and arrangement of EC are affected by the regulation of matrix morphology and hardness	[92]

Halfsphere					
Material	Cell type	Spacing	Height	Findings	Ref.
PLGA	RAEC	195.3 $nm$ 312.5 $nm$ 410.2 $nm$ 957 $nm$	4.9 $nm$ 86.3 $nm$ 18.9 $nm$ 396.4 $nm$	The vertical dimension of the structural surface has the greatest impact on cell adhesion	[96]

Circular cone						
Material	Cell type	Spacing	Height	Tip diameter	Findings	Ref.
Si	EC	6 $\mu m$	10 $\mu m$	0.15 $\mu m$	Local contact with mature and matrix fiber formation is inhibited	[73]
PEG	P19 EC	150 - 500 $nm$	250- 300 $nm$	–	The influence of nano-morphology on the surface adsorption site	[42]

*Cells (*HGTFN* endothelial cell isolated from a human tendon granuloma)

The shape of nanoislands can also be found in aperiodic micro-nanostructures, but there are periodic differences from those mentioned here. However, the overall roughness and the law of impact on cells are similar to aperiodic structures. For example, using microphase separation of the copolymers of PS and PbrS, nanoscale island structures with different heights were produced, and an EC with a 13-nm-high island was the longest when using staining observation. Using SEM, the interaction between cellular filamentous pseudopodia as one part of the cytoskeleton and the structure was determined. The results demonstrated the acceleration of cell proliferation, and the cell in the 13-nm-high island was the earliest to react, indicating the different response times of the cell or different regulatory intensity. This proves the importance of pseudopodia in affecting the cell. Contrary to PS, EC showed a significantly more dispersed morphology on PBrS, which is believed to be due to the better hydrophilicity of PBrS, illustrating the impact of material surface properties once again [95]. Although it is generally believed that hydrophilic materials are more conducive to cell growth and adhesion, there is no linear relationship. Therefore, the exact law of the effect of wettability on cells still needs to be tested.

Kim et al. also promoted EC adhesion through a conical structure, which was attributed to the increase in the area where cells can adhere and the change in the binding sites of cells due to nano-morphology (Fig. 6b) [42]. Carpenter et al. introduced the factor of surface energy, believing that the adsorption of serum proteins caused by the surface's free energy is the reason for the increased adhesion of EC [96]. Various studies have tried to summarize the role of micro-nanostructures to uncover the rules of their interaction with cells. However, the surface energy, stiffness, etc., do not follow a specific law due to the interweaving of various factors. Therefore, the most recent research has returned to the characteristic size of the structure itself. This is also a fundamental change in micro–nanostructures, and surface energy, wettability, and stiffness are all affected by it. However, the influence of characteristic sizes of different structures on cells has different rules, making it difficult to form a unified result for the experiment.

In most cases, two-dimensional periodic micro-nanostructures still mainly affect cell adhesion and fail to exhibit unique effects compared to non-periodic micro–nanostructures. However, the effect of microcolumns can also cause cell alignment, which forces us to re-examine the reasons for cell alignment. Last but not least, researchers have not created a large-spanning structure. Thus, whether the function of the structure can be found in the specific interaction between cells directly is unclear, and many follow-up experiments on the intensity and regularity of the impact on cells are needed.

## Discussion

When preparing micro–nanostructures, even using simple methods, many solid structures can be prepared by combining processes, selecting different materials, and controlling the size. In combination with traditional cell-detection methods and adjustment based on structural conditions, many studies have achieved basic regulation of the morphology, proliferation, adhesion, and orientation of ECs and SMCs in vitro. However, due to the different materials and processes used, these regulatory laws do not have consistency. Generally, however, there is a regulatory peak with changes in structural size. Therefore, simple predictions can be made of the functions of structures with different parameters, provided that the materials and surface treatment processes are not changed, which also leads to significant limitations. Overall, the promotion or inhibition of cells and their structural effects are related to the biocompatibility of the material itself. The most widely used and effective methods are one-dimensional periodic structure grooves, which are mainly reflected in cell shape elongation and control of the cell arrangement.

While non-periodic micro-nanostructures and two-dimensional periodic structures have the most significant effects on the adhesion of cells, only the microcolumn structure impacts cell alignment. Although the anisotropy of one-dimensional periodic structures is considered to be a key factor in their regulation of cell alignment and growth, it is not possible to explain the effect of two-dimensional microcylinder structures or the relationship between the microcylinder and groove. Moreover, experimental research focused on ECs and SMCs is still limited, and the roughness and wettability of cells cannot be used to uncover the functional rules of the structure. It is possible that this feature parameter masks certain features because different micro-nanostructures can have the same wettability or roughness.

Research on the intrinsic mechanism mainly starts with substances such as actin, focal adhesion kinase, and fibronectin, which are the main substances involved in traditional theories. These are also the fundamental factors affecting cell interactions because an increased secretion of proteins increases the whole concentration, affecting all cells. However, this explanation mainly focuses on the structural morphology that changes the distribution of proteins and adhesion sites, thereby affecting the adhesion, proliferation, and arrangement of cells. However, internal vascular diseases involve changes in many proteins, and whether these key proteins can reflect all issues is a question that needs further consideration in vascular stent research. In addition, the coating and modification of the structural surface change in the chemical groups, wettability, and biocompatibility must be studied, along with the relationship between the material and cell, to consider the change in intrinsic properties.

As for the structure alone, it is necessary to exclude the interference of these factors to determine the regularity of the effects of micro-nanostructures on cells. However, current research experiments lack repetition, and there are differences in research findings that cannot fully explain cell behavior. With regard to the goal of achieving rapid endothelialization and reducing intravascular restenosis of vascular stents, although a specific and regular explanation cannot be obtained, the currently studied structures have achieved basic effects on ECs and SMCs in vitro, which indicates the progress of research on the application of micro–nanostructures in vascular stents and lays the foundation for its application.

With the help of these studies, micro–nanostructured composite stents are expected to become a novel approach for treating vascular diseases, as they can achieve rapid endothelialization or reduce vascular restenosis, similar to DES. The most important result is that they do not cause drug-induced side effects, and there are no population-adaptation issues, such as allergies. In addition, micro–nanostructure can reflect cell specificity and simultaneously promote ECs and inhibit SMCs, which is currently not achieved by commercial DES. The series of rules discovered by researchers can provide guidance for the selection of materials, structural shapes, and sizes. Crucially this effect has stability and permanence. However, the promotion or inhibition effect of micro–nanostructure on cells has always been limited. For some critically ill patients, it may be more appropriate to combine drugs, balancing the side effects of drugs through physical signals.

Finally, biodegradable stents can also utilize micro–nanostructure to achieve better results without affecting their degradation performance. Therefore, combining micro–nanostructure and vascular stents can improve stent function without causing side effects and may be a main trend in future research.

## Conclusion

According to the regulation of EC and SMC by micro–nanostructure, the paper briefly describes the preparation methods of micro–nanostructure and cell experiments used in previous studies. Meanwhile, based on the periodic classification of micro–nanostructures, the functions of them were analyzed. Although the influence of micro–nanostructure on cell proliferation, adhesion and other behaviors is not as strong as that of drugs, they have unique advantages in guiding and regulating cell distribution and orientation. The functionality of vascular stent will be further improved if micro–nanostructure can be applied. In addition, we believe it will have more advantages in fields such as wound healing and scar repair if the detailed laws of cellular regulation by micro–nanostructure can be obtained.

## Data Availability

No datasets were generated or analyzed during the current study.
